# Supplementary material for: Vasodilating Effects of Antispasmodic Agents and Their Cytotoxicity in Vascular Smooth Muscle Cells and Endothelial Cells—Potential Application in Microsurgery
Source: Int J Mol Sci. 2023 Jun 29;24(13):10850. doi: 10.3390/ijms241310850 (PMC10341634; doi:10.3390/ijms241310850)
Supplement: Supplementary file 1 [file ijms-24-10850-s001.zip › ijms-2450925-supplementary.pptx]

## Slide 1
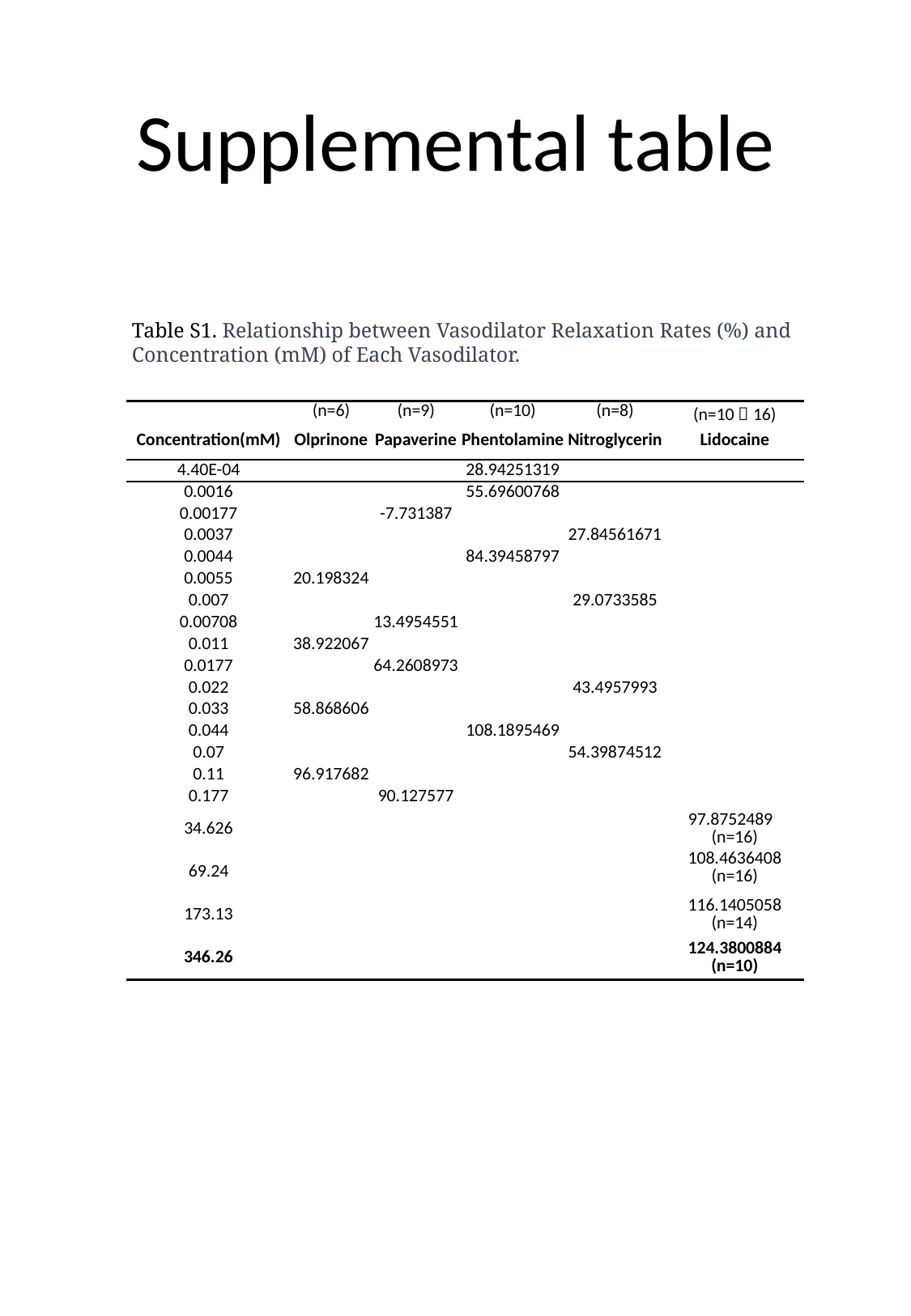

Supplemental table
Table S1. Relationship between Vasodilator Relaxation Rates (%) and Concentration (mM) of Each Vasodilator.
| | (n=6) | (n=9) | (n=10) | (n=8) | (n=10～16) |
| --- | --- | --- | --- | --- | --- |
| Concentration(mM) | Olprinone | Papaverine | Phentolamine | Nitroglycerin | Lidocaine |
| 4.40E-04 | | | 28.94251319 | | |
| 0.0016 | | | 55.69600768 | | |
| 0.00177 | | -7.731387 | | | |
| 0.0037 | | | | 27.84561671 | |
| 0.0044 | | | 84.39458797 | | |
| 0.0055 | 20.198324 | | | | |
| 0.007 | | | | 29.0733585 | |
| 0.00708 | | 13.4954551 | | | |
| 0.011 | 38.922067 | | | | |
| 0.0177 | | 64.2608973 | | | |
| 0.022 | | | | 43.4957993 | |
| 0.033 | 58.868606 | | | | |
| 0.044 | | | 108.1895469 | | |
| 0.07 | | | | 54.39874512 | |
| 0.11 | 96.917682 | | | | |
| 0.177 | | 90.127577 | | | |
| 34.626 | | | | | 97.8752489 (n=16) |
| 69.24 | | | | | 108.4636408 (n=16) |
| 173.13 | | | | | 116.1405058 (n=14) |
| 346.26 | | | | | 124.3800884 (n=10) |

## Slide 2
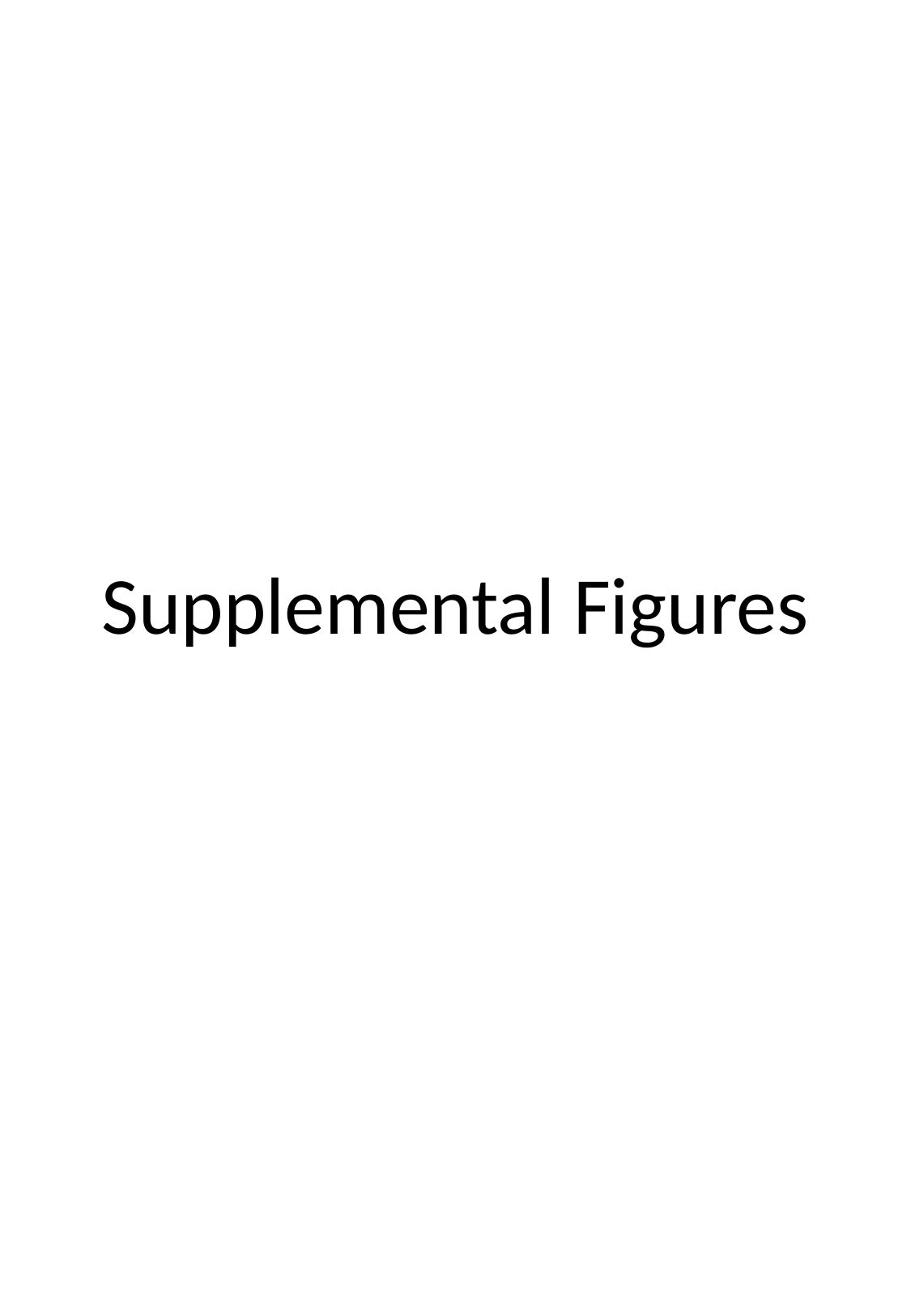

Supplemental Figures

## Slide 3
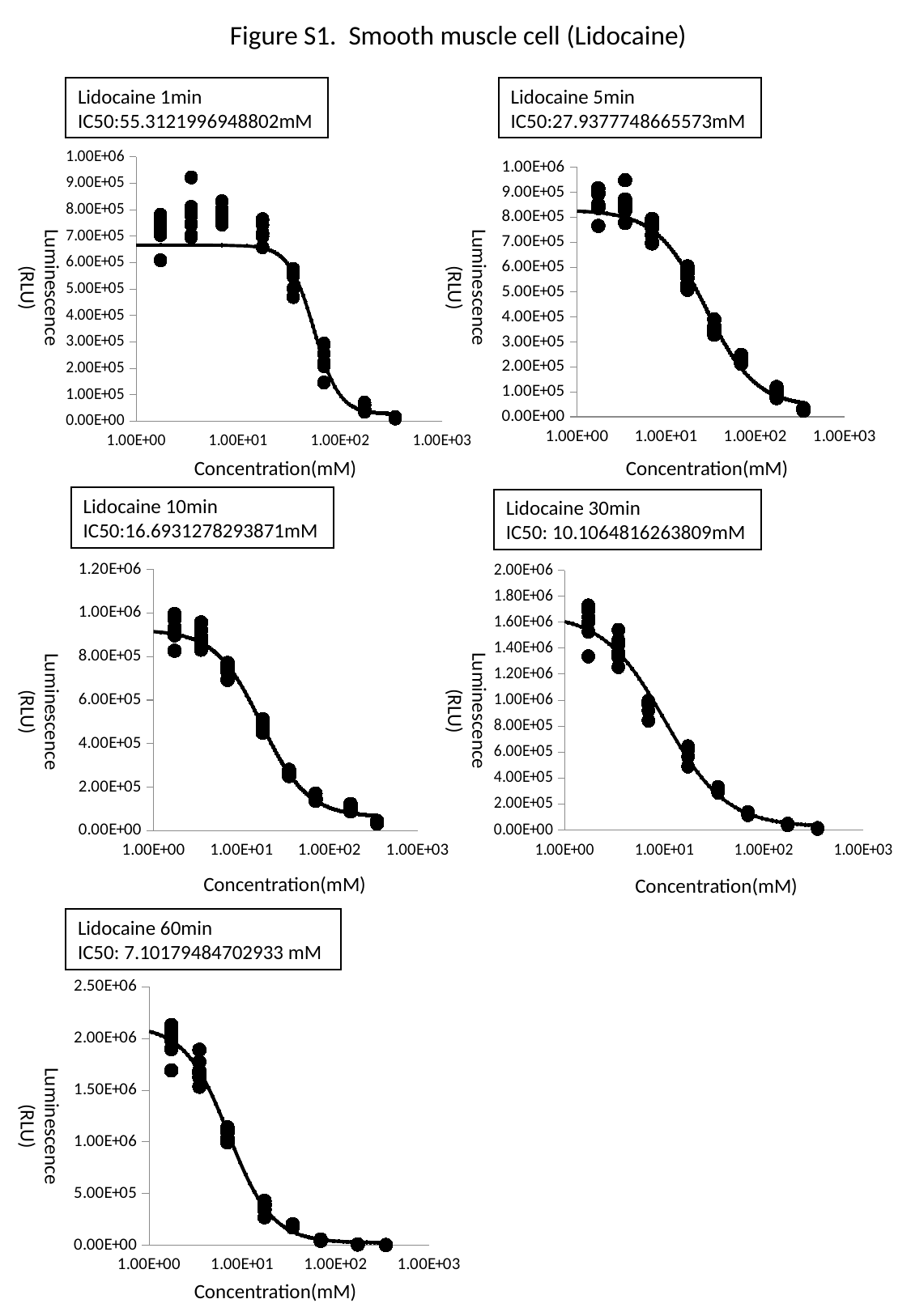

Figure S1. Smooth muscle cell (Lidocaine)
Lidocaine 5min
IC50:27.9377748665573mM
Lidocaine 1min
IC50:55.3121996948802mM
### Chart
| Category | | |
|---|---|---|
### Chart
| Category | | |
|---|---|---|Luminescence (RLU)
Luminescence (RLU)
Concentration(mM)
Concentration(mM)
Lidocaine 10min
IC50:16.6931278293871mM
Lidocaine 30min
IC50: 10.1064816263809mM
### Chart
| Category | | |
|---|---|---|
### Chart
| Category | | |
|---|---|---|Luminescence (RLU)
Luminescence (RLU)
Concentration(mM)
Concentration(mM)
Lidocaine 60min
IC50: 7.10179484702933 mM
### Chart
| Category | | |
|---|---|---|Luminescence (RLU)
Concentration(mM)

## Slide 4
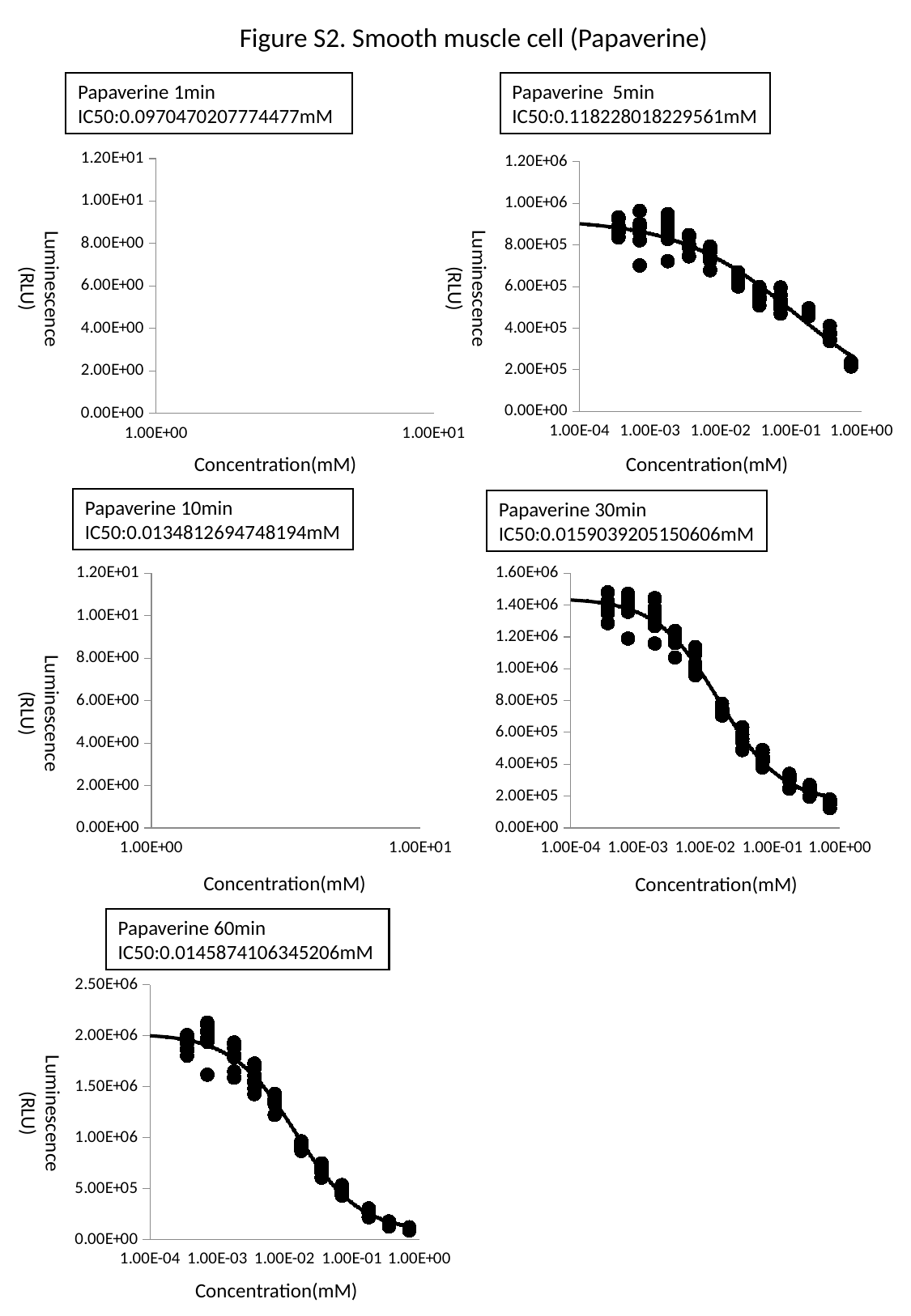

Figure S2. Smooth muscle cell (Papaverine)
Papaverine 5min
IC50:0.118228018229561mM
Papaverine 1min
IC50:0.0970470207774477mM
### Chart
| Category | | |
|---|---|---|
### Chart
| Category | | |
|---|---|---|Luminescence (RLU)
Luminescence (RLU)
Concentration(mM)
Concentration(mM)
Papaverine 10min
IC50:0.0134812694748194mM
Papaverine 30min
IC50:0.0159039205150606mM
### Chart
| Category | | |
|---|---|---|
### Chart
| Category | | |
|---|---|---|Luminescence (RLU)
Concentration(mM)
Concentration(mM)
Papaverine 60min
IC50:0.0145874106345206mM
### Chart
| Category | | |
|---|---|---|Luminescence (RLU)
Concentration(mM)

## Slide 5
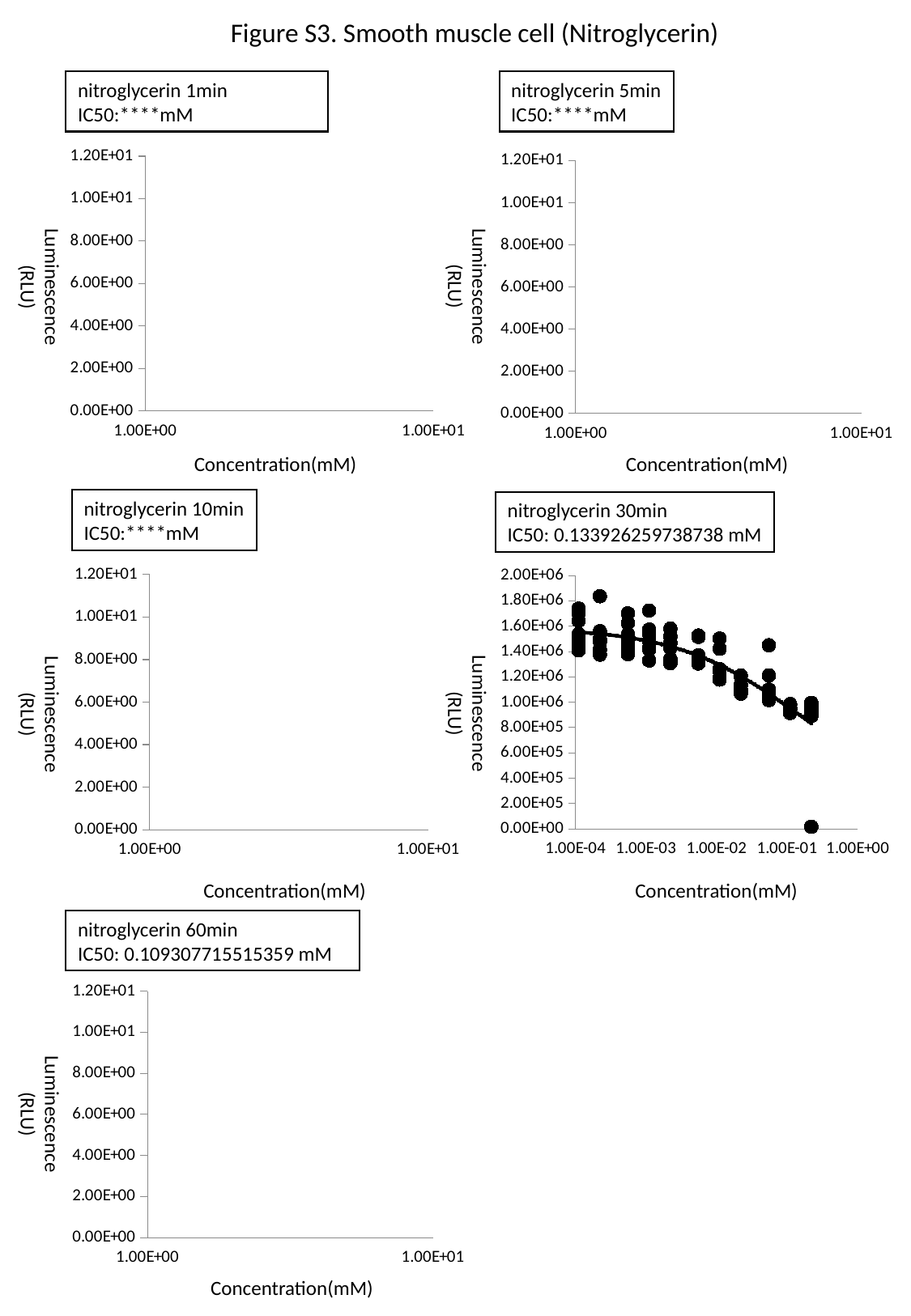

Figure S3. Smooth muscle cell (Nitroglycerin)
nitroglycerin 5min
IC50:****mM
nitroglycerin 1min
IC50:****mM
### Chart
| Category | | |
|---|---|---|
### Chart
| Category | | |
|---|---|---|Luminescence (RLU)
Luminescence (RLU)
Concentration(mM)
Concentration(mM)
nitroglycerin 10min
IC50:****mM
nitroglycerin 30min
IC50: 0.133926259738738 mM
### Chart
| Category | | |
|---|---|---|
### Chart
| Category | | |
|---|---|---|Luminescence (RLU)
Luminescence (RLU)
Concentration(mM)
Concentration(mM)
nitroglycerin 60min
IC50: 0.109307715515359 mM
### Chart
| Category | | |
|---|---|---|Luminescence (RLU)
Concentration(mM)

## Slide 6
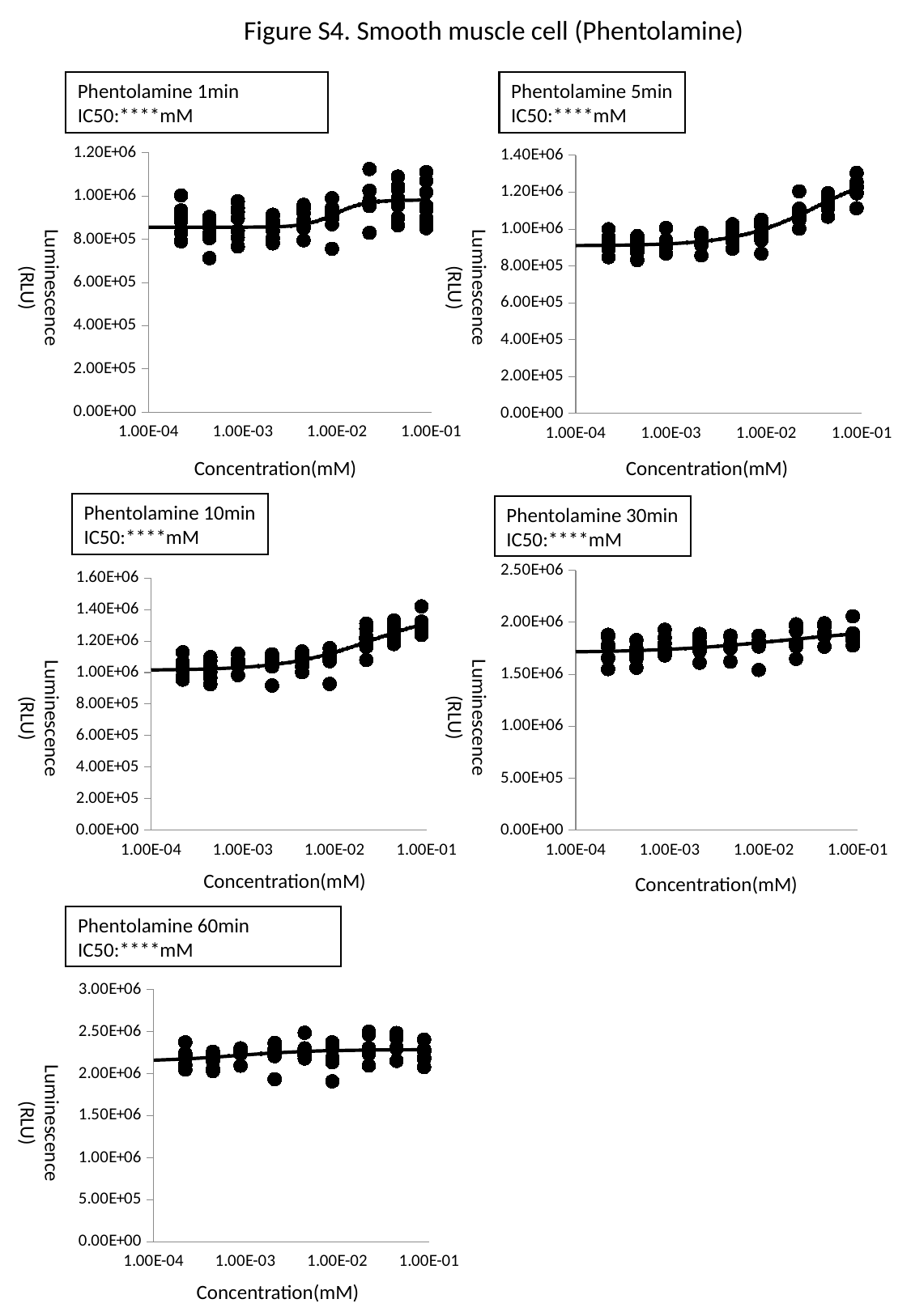

Figure S4. Smooth muscle cell (Phentolamine)
Phentolamine 1min
IC50:****mM
Phentolamine 5min
IC50:****mM
### Chart
| Category | | |
|---|---|---|
### Chart
| Category | | |
|---|---|---|Luminescence (RLU)
Luminescence (RLU)
Concentration(mM)
Concentration(mM)
Phentolamine 10min
IC50:****mM
Phentolamine 30min
IC50:****mM
### Chart
| Category | | |
|---|---|---|
### Chart
| Category | | |
|---|---|---|Luminescence (RLU)
Luminescence (RLU)
Concentration(mM)
Concentration(mM)
Phentolamine 60min
IC50:****mM
### Chart
| Category | | |
|---|---|---|Luminescence (RLU)
Concentration(mM)

## Slide 7
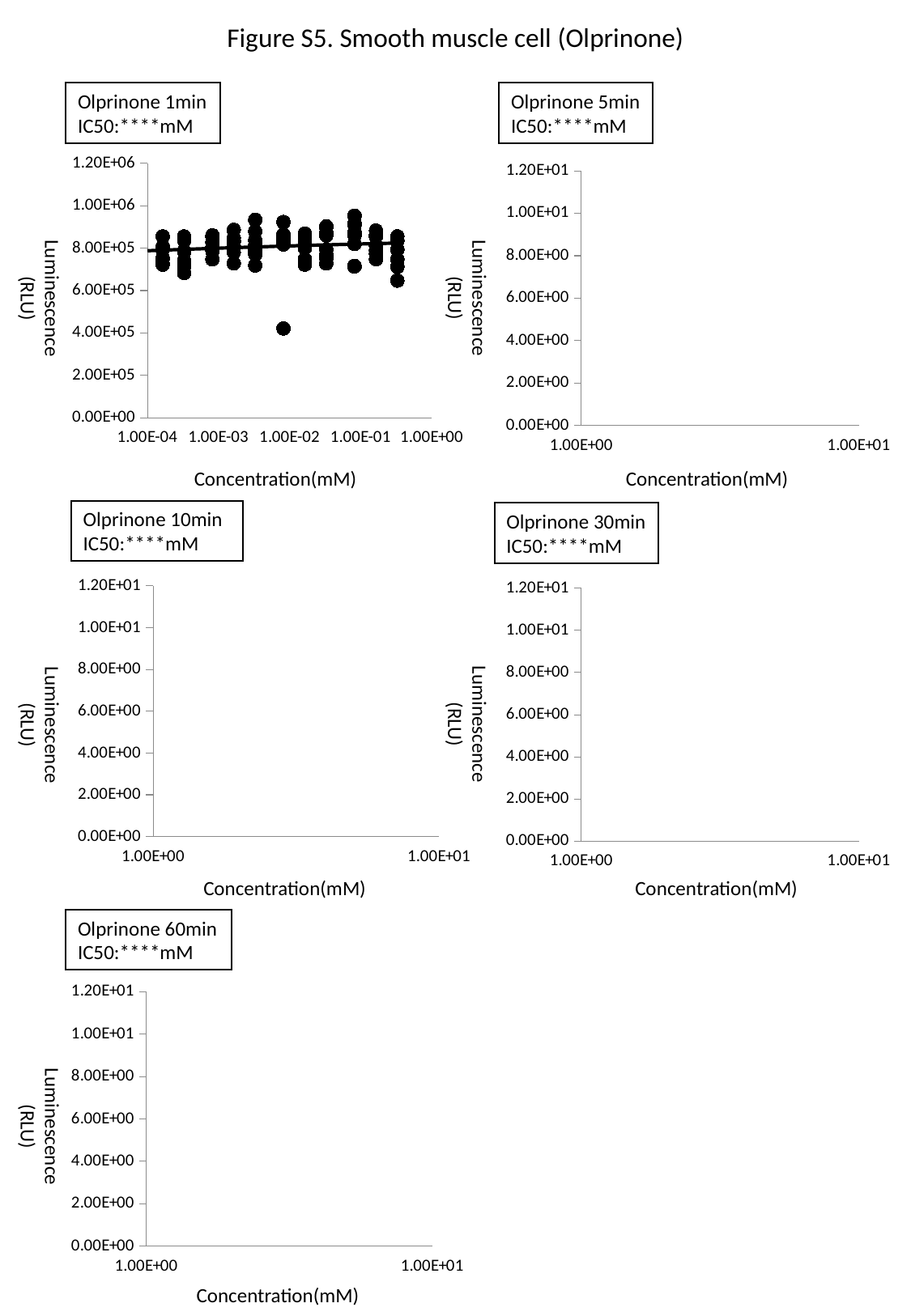

Figure S5. Smooth muscle cell (Olprinone)
Olprinone 5min
IC50:****mM
Olprinone 1min
IC50:****mM
### Chart
| Category | | |
|---|---|---|
### Chart
| Category | | |
|---|---|---|Luminescence (RLU)
Luminescence (RLU)
Concentration(mM)
Concentration(mM)
Olprinone 10min
IC50:****mM
Olprinone 30min
IC50:****mM
### Chart
| Category | | |
|---|---|---|
### Chart
| Category | | |
|---|---|---|Luminescence (RLU)
Luminescence (RLU)
Concentration(mM)
Concentration(mM)
Olprinone 60min
IC50:****mM
### Chart
| Category | | |
|---|---|---|Luminescence (RLU)
Concentration(mM)

## Slide 8
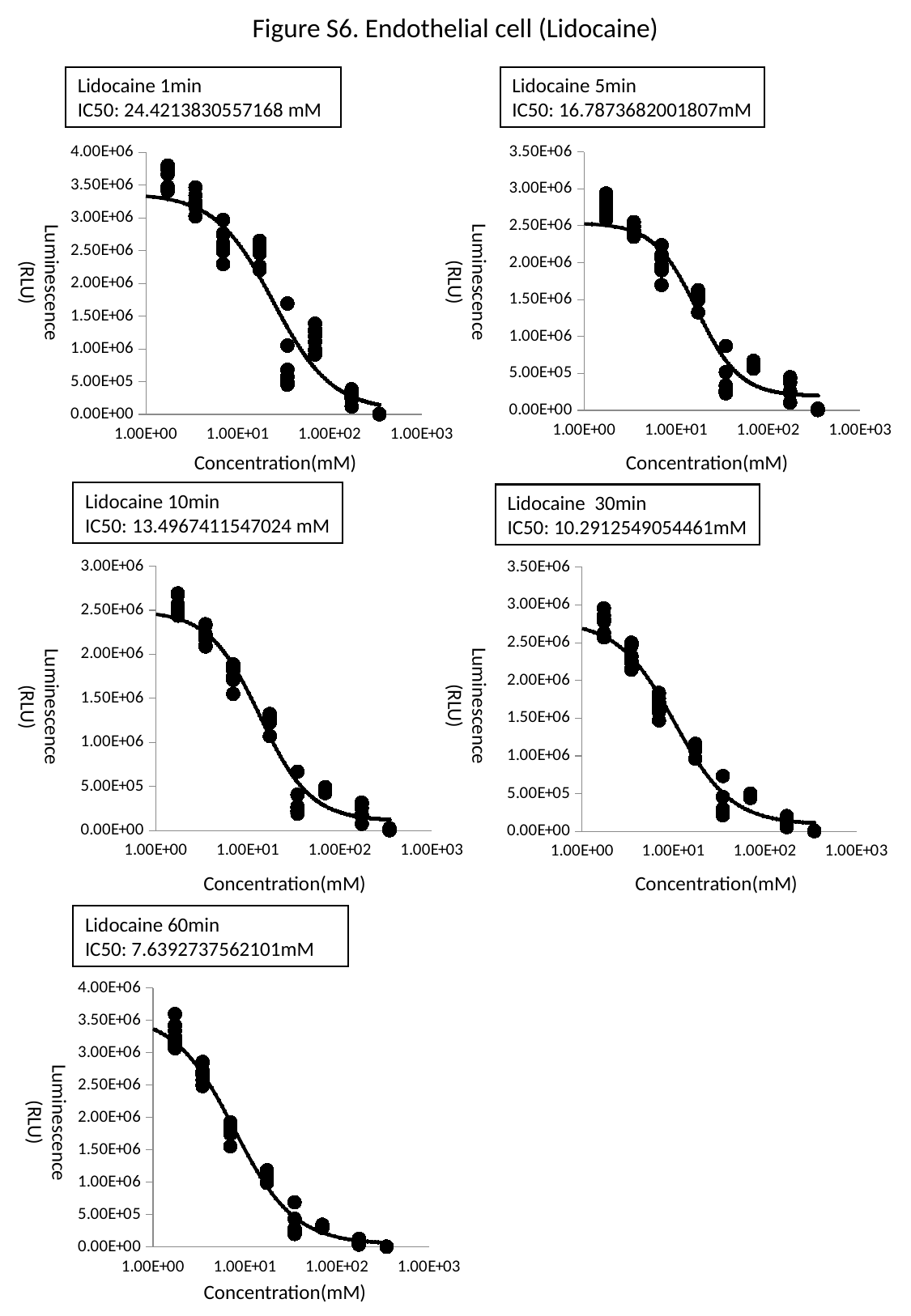

Figure S6. Endothelial cell (Lidocaine)
Lidocaine 1min
IC50: 24.4213830557168 mM
Lidocaine 5min
IC50: 16.7873682001807mM
### Chart
| Category | | |
|---|---|---|
### Chart
| Category | | |
|---|---|---|Luminescence (RLU)
Luminescence (RLU)
Concentration(mM)
Concentration(mM)
Lidocaine 10min
IC50: 13.4967411547024 mM
Lidocaine 30min
IC50: 10.2912549054461mM
### Chart
| Category | | |
|---|---|---|
### Chart
| Category | | |
|---|---|---|Luminescence (RLU)
Luminescence (RLU)
Concentration(mM)
Concentration(mM)
Lidocaine 60min
IC50: 7.6392737562101mM
### Chart
| Category | | |
|---|---|---|Luminescence (RLU)
Concentration(mM)

## Slide 9
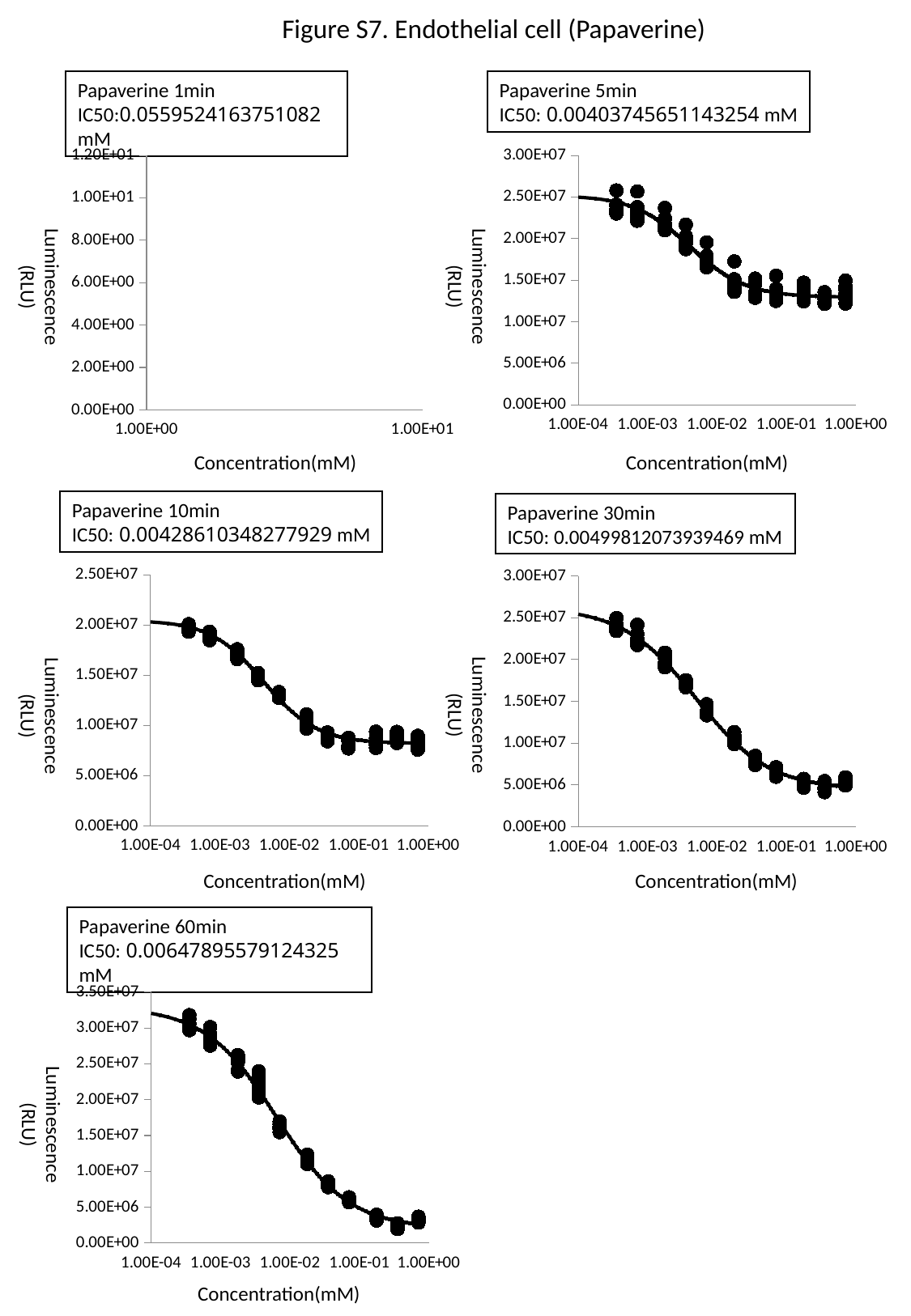

Figure S7. Endothelial cell (Papaverine)
Papaverine 1min
IC50:0.0559524163751082mM
Papaverine 5min
IC50: 0.00403745651143254 mM
### Chart
| Category | | |
|---|---|---|
### Chart
| Category | | |
|---|---|---|Luminescence (RLU)
Luminescence (RLU)
Concentration(mM)
Concentration(mM)
Papaverine 10min
IC50: 0.00428610348277929 mM
Papaverine 30min
IC50: 0.00499812073939469 mM
### Chart
| Category | | |
|---|---|---|
### Chart
| Category | | |
|---|---|---|Luminescence (RLU)
Luminescence (RLU)
Concentration(mM)
Concentration(mM)
Papaverine 60min
IC50: 0.00647895579124325 mM
### Chart
| Category | | |
|---|---|---|Luminescence (RLU)
Concentration(mM)

## Slide 10
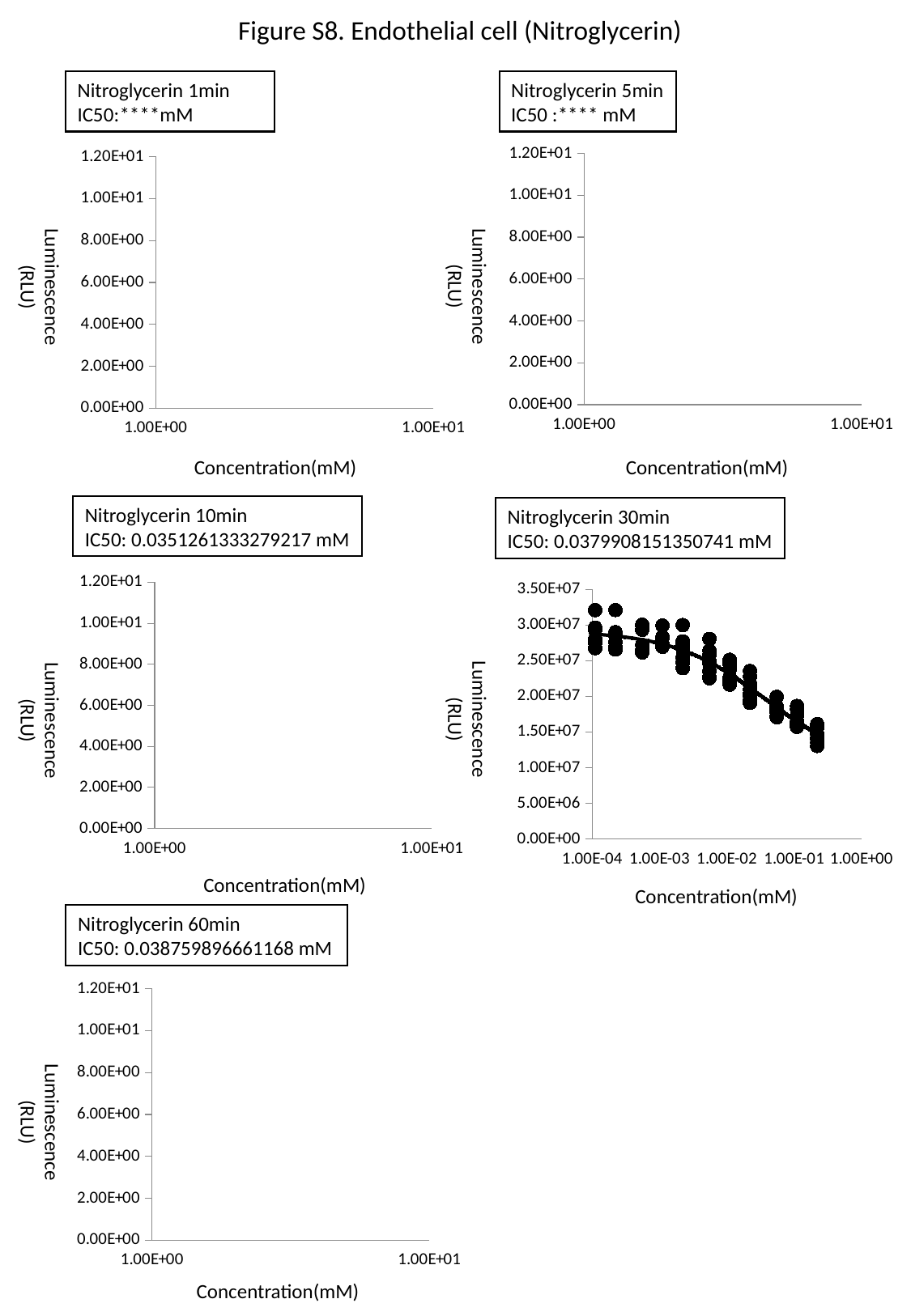

Figure S8. Endothelial cell (Nitroglycerin)
Nitroglycerin 1min
IC50:****mM
Nitroglycerin 5min
IC50 :**** mM
### Chart
| Category | | |
|---|---|---|
### Chart
| Category | | |
|---|---|---|Luminescence (RLU)
Luminescence (RLU)
Concentration(mM)
Concentration(mM)
Nitroglycerin 10min
IC50: 0.0351261333279217 mM
Nitroglycerin 30min
IC50: 0.0379908151350741 mM
### Chart
| Category | | |
|---|---|---|
### Chart
| Category | | |
|---|---|---|Luminescence (RLU)
Luminescence (RLU)
Concentration(mM)
Concentration(mM)
Nitroglycerin 60min
IC50: 0.038759896661168 mM
### Chart
| Category | | |
|---|---|---|Luminescence (RLU)
Concentration(mM)

## Slide 11
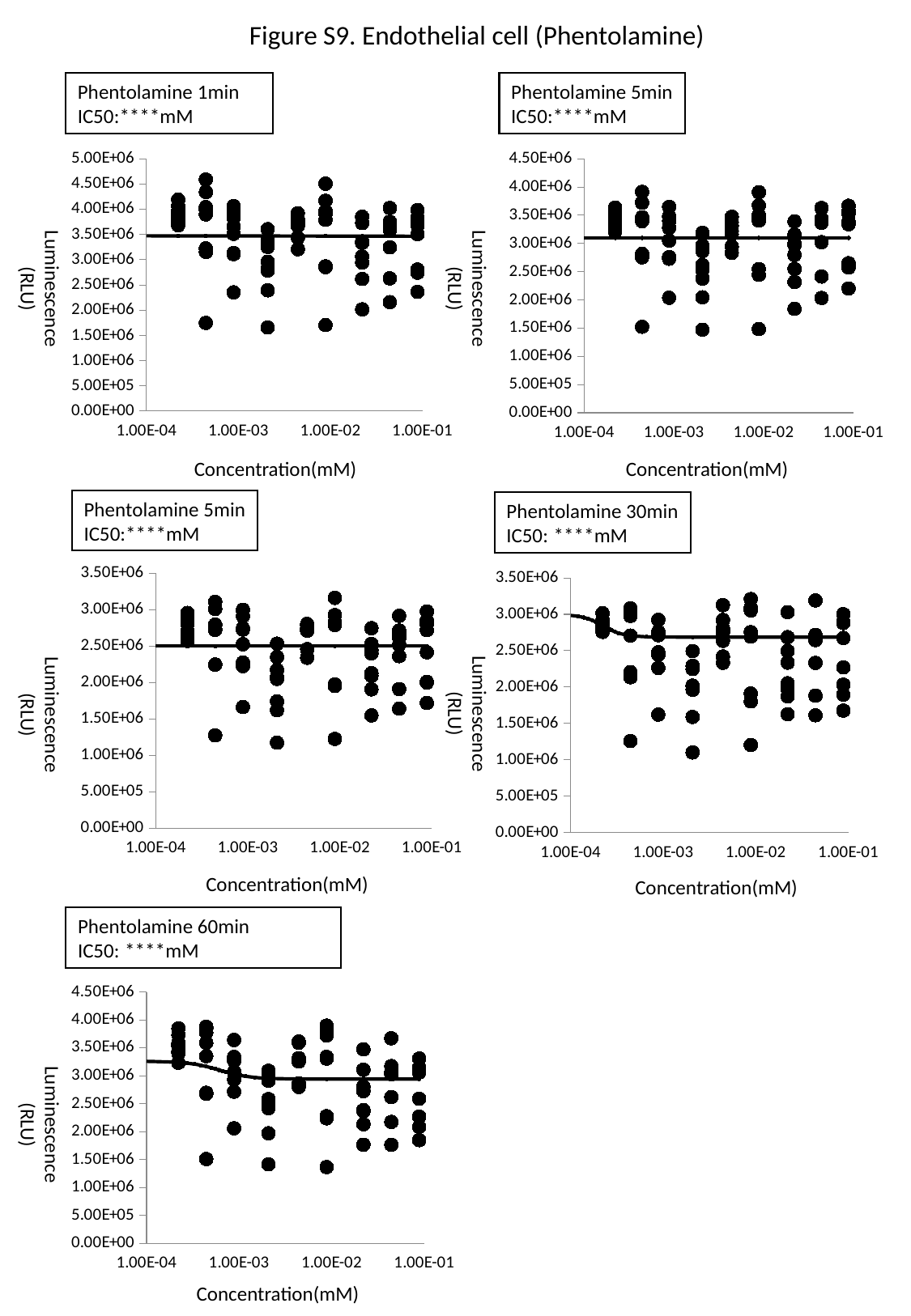

Figure S9. Endothelial cell (Phentolamine)
Phentolamine 1min
IC50:****mM
Phentolamine 5min
IC50:****mM
### Chart
| Category | | |
|---|---|---|
### Chart
| Category | | |
|---|---|---|Luminescence (RLU)
Luminescence (RLU)
Concentration(mM)
Concentration(mM)
Phentolamine 5min
IC50:****mM
Phentolamine 30min
IC50: ****mM
### Chart
| Category | | |
|---|---|---|
### Chart
| Category | | |
|---|---|---|Luminescence (RLU)
Luminescence (RLU)
Concentration(mM)
Concentration(mM)
Phentolamine 60min
IC50: ****mM
### Chart
| Category | | |
|---|---|---|Luminescence (RLU)
Concentration(mM)

## Slide 12
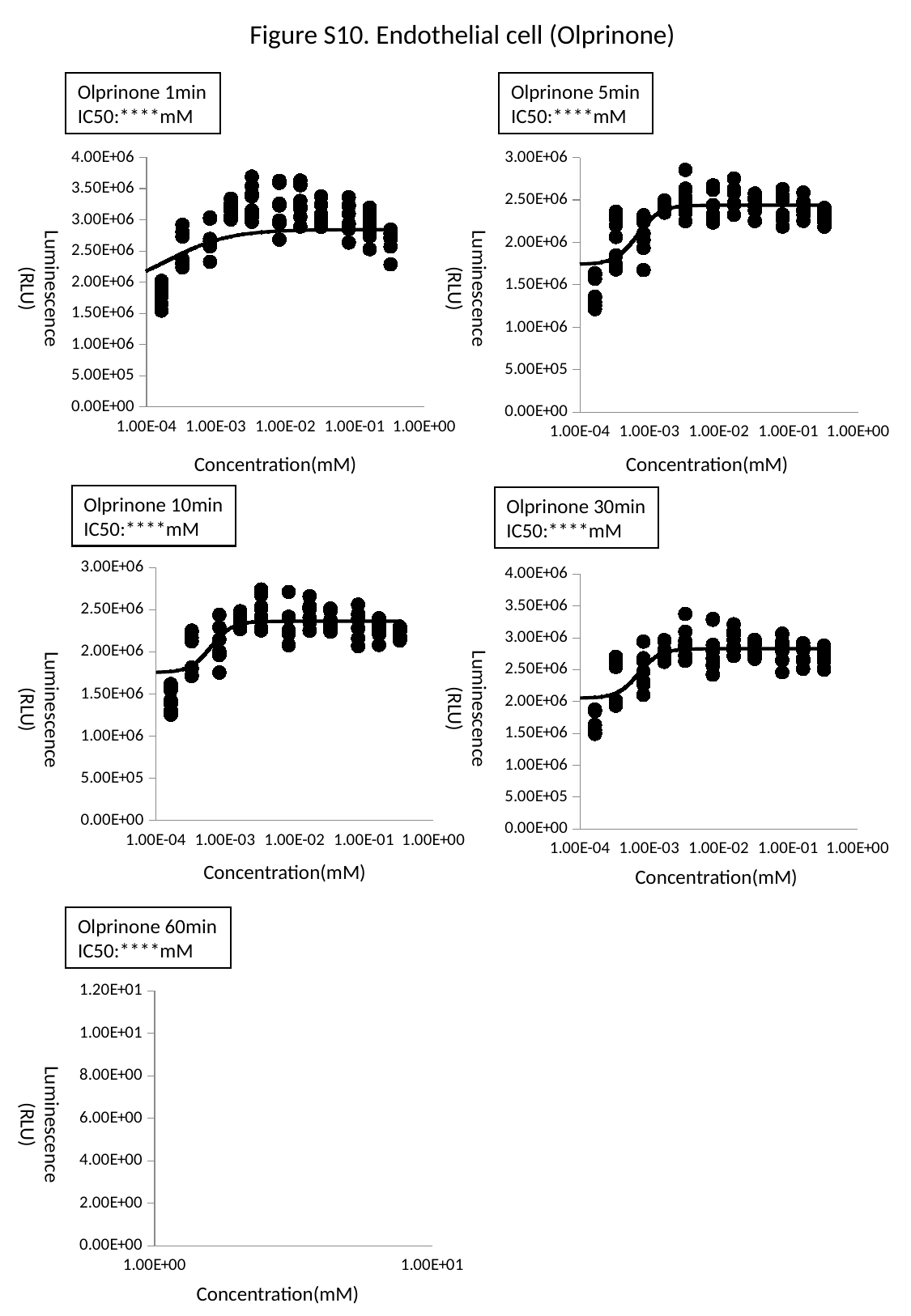

Figure S10. Endothelial cell (Olprinone)
Olprinone 1min
IC50:****mM
Olprinone 5min
IC50:****mM
### Chart
| Category | | |
|---|---|---|
### Chart
| Category | | |
|---|---|---|Luminescence (RLU)
Luminescence (RLU)
Concentration(mM)
Concentration(mM)
Olprinone 10min
IC50:****mM
Olprinone 30min
IC50:****mM
### Chart
| Category | | |
|---|---|---|
### Chart
| Category | | |
|---|---|---|Luminescence (RLU)
Luminescence (RLU)
Concentration(mM)
Concentration(mM)
Olprinone 60min
IC50:****mM
### Chart
| Category | | |
|---|---|---|Luminescence (RLU)
Concentration(mM)
